# Extra Climate Benefits From Afforestation Due to Reduced Forest Fragmentation in China

**DOI:** 10.1002/advs.75872

**Published:** 2026-06-15

**Authors:** Nan Meng, Wei Li, Philippe Ciais, Martin Brandt, Xiaowei Tong, Yanzheng Yang, Guofeng Shen, Wei Du, Lei Zhu, Yi Leng, Minxuan Sun, Mengjie Han, Xuemei Zhang, Jie Zhang, Nan Wang, Dan Tong, Chao Wu, Wenhao Dong, Shu Tao, Bo Pan

**Affiliations:** ^1^ Department of Earth System Science Ministry of Education Key Laboratory for Earth System Modeling Institute For Global Change Studies Tsinghua University Beijing China; ^2^ Ministry of Education Ecological Field Station For East Asian Migratory Birds Department of Earth System Science Tsinghua University Beijing China; ^3^ Laboratoire des Sciences du Climat et de l'Environnement LSCE/IPSL CEA‐CNRS‐UVSQ Université Paris‐Saclay Gif‐sur‐Yvette France; ^4^ Department of Geosciences and Natural Resource Management University of Copenhagen Copenhagen Denmark; ^5^ Key Laboratory for Agro‐ecological Processes in Subtropical Region Institute of Subtropical Agriculture Chinese Academy of Sciences Changsha China; ^6^ State Key Laboratory of Regional and Urban Ecology Research Center for Eco‐Environmental Sciences Chinese Academy of Sciences Beijing China; ^7^ University of Chinese Academy of Sciences Beijing China; ^8^ College of Urban and Environmental Sciences Peking University Beijing China; ^9^ Institute of Carbon Neutrality Peking University Beijing China; ^10^ Faculty of Environmental Science & Engineering Kunming University of Science & Technology Kunming China; ^11^ Sino‐French Institute for Earth System Science Peking University Beijing China

**Keywords:** afforestation, biogeochemical cycle, biophysical process, climate effects, edge effects, forest fragmentation

## Abstract

Afforestation connects isolated forests into larger contiguous forests, reducing forest fragmentation. This process decreases edge areas by transforming edge forests into new interior forests (termed transformed forests). However, the extra climate benefits from edge reductions in transformed forests, beyond those provided by the planted forests themselves, remain unclear. Here, CO_2_ sequestration from increased biomass (biogeochemical effect) and emissions from decreased albedo (biophysical effect) of transformed forests in China are estimated, using multiple high‐resolution remote‐sensing datasets. The planted forest area (89.6 M ha) accounted for 35.5% of China's forest area in 2015, transforming 51.8 M ha of edge forests into interior forests. A cumulative increase of 1.4±0.2 Pg CO_2_e in the transformed forests is found, compared with a biomass increase of 10.3±0.4 Pg CO_2_e in the planted forests over ~1980–2015. These transformed forests also induce a biophysical warming effect of −0.9 Pg CO_2_e, partially offsetting the cooling effect from increased biomass. Combining both effects, transformed forests provide a net CO_2_e gain of 0.5±0.2 Pg CO_2_e, representing an extra 6.6±2.7% of the direct climate benefits from planted forests. This study reveals previously ignored extra climate benefits from reduced forest fragmentation alongside forest expansion, offering new perspectives on mitigating climate warming through afforestation.

## Graphical abstract

Afforestation in China reduced forest fragmentation in 2015, transforming 51.8 M ha of edge forests into interior forests. This enhanced carbon sequestration (1.4±0.2 Pg CO_2_e, a cooling biogeochemical effect), while reduced albedo (−0.9 Pg CO_2_e, a warming biophysical effect) partially offset the gain, yielding a net extra climate benefit of approximately 6.6±2.7% beyond the direct effects of planted forests alone.

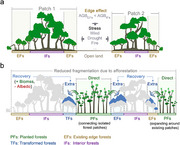

## 1 Introduction

Forests influence global climate through both biogeochemical (e.g., carbon sequestration) and biophysical (e.g., surface energy balance) processes [1], with net effects depending on their interplay between these processes [2]. Beyond forest area, the spatial configuration of forests, particularly fragmentation, also plays a key role in shaping these climate impacts [3]. Forest fragmentation caused by deforestation transforms interior forests into edge forests, leading, as observed in tropical forests, to biomass carbon loss and local land surface cooling [4, 5]. Edge forests exhibit lower aboveground biomass than interior forests across approximately 97% of global forests, with an average reduction of 16% [6]. Moreover, edge forests are consistently warmer than interior forests, with variations across biomes and seasons [7]. These patterns occur because forest edges typically experience stronger wind, more intense drought, higher fire risk, and greater biotic disturbances [6]. Consequently, in addition to the forest cover loss, the edge‐related biogeochemical and local biophysical changes are receiving increasing attention.

Afforestation, conversely, can alleviate forest fragmentation by connecting isolated forest patches into larger and contiguous forests and converting edge forests into interior forests [8, 9], hereafter referred to as “transformed forests”. In China, a decline in forest fragmentation due to large‐scale afforestation was observed between 2000 and 2020 [10]. Edge‐to‐interior transformations may alleviate edge effects [11], contributing to the recovery of former edge zones (Figure S1). While these transformations can reduce local warming at forest edges, their primary contribution is to global climate mitigation by increasing biomass accumulation and altering surface albedo, expressed in terms of CO_2_‐equivalent (CO_2_e) reductions. These biogeochemical and biophysical benefits go beyond those resulting from the simple expansion of newly planted forest area, hereafter referred to as “extra climate benefits”. However, the magnitude and spatial distribution of these extra climate benefits arising from reduced forest fragmentation remain poorly quantified.

Here, we quantify the extra climate benefits of transformed forests on both biogeochemical and biophysical effects using a space‐for‐time method (Figure S1). Specifically, we first calculate changes in forest fragmentation and identify the transformed forests affected by new afforestation using a high‐resolution forest management map [12]. We then estimate the biomass increase within these transformed forests with their paired existing edge forests based on multiple satellite‐derived aboveground biomass (AGB) datasets [13, 14, 15], and then convert it to CO_2_e changes. Next, we quantify the associated albedo decreases using MCD43A3 C6.1 surface albedo data [16] and translate these into CO_2_e terms. For comparison, we also assess the direct biogeochemical and biophysical effects of newly planted forests, defined as “direct climate benefits” (Figures S2 and S3).

## 2 Results

### 2.1 Changes in Forest Fragmentation Due to Afforestation

**FIGURE 1:**
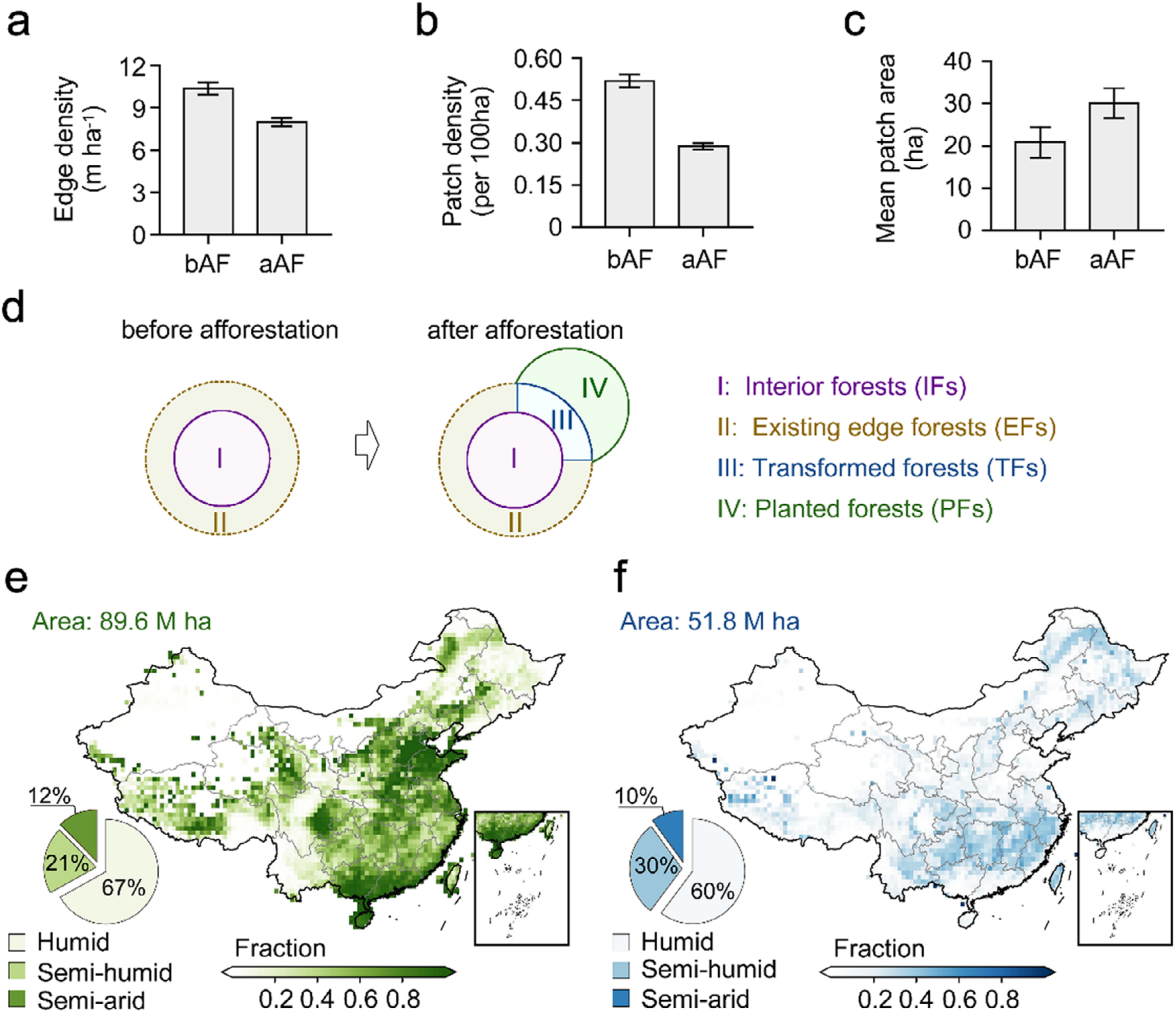
Changes in forest fragmentation by afforestation and forest types in China. (a–c), Forest fragmentation index before afforestation (bAF) and after afforestation (aAF). The bar plots with error bars represent the mean and 95% confidence interval for edge density (a), patch density (b), and mean patch area (c). (d) Schematic diagram for different forest types (see details in Figure S1). Dashed lines represent edges in the 1980s, and solid lines represent edges in 2015. (e,f), Spatial distribution of the fraction of planted forests since ~1980 (e) and transformed forests (f), calculated as the proportion of each forest type relative to the total forest area within each 50 km × 50 km grid cell. The pie plots in (e) and (f) show the area proportion of PFs and TFs in humid, semi‐humid, and semi‐arid regions. See Figure S24 for detailed information on the spatial distribution of aridity.

According to the forest management dataset [12], the area of natural forests in China in 2015 was 162.4 million hectares (M ha). Planted forests (PFs) covered 89.6 M ha, thus accounting for 35.5% of the total forest area. These estimates are generally consistent with national inventory data and other remote‐sensing estimates (Text S1, Table S4). The number of forest patches has decreased from 4.2 million before afforestation (prior to ~1980) to 2.4 million after afforestation (by 2015) (Text S2, Table S5). As a result, the degree of forest fragmentation has been greatly reduced, as indicated by a reduction in edge density (−2.4 m ha^−1^), a decrease in patch density (−0.2 per 100 ha), and an increase in mean patch size (+9.2 ha) (Figure 1a–c). The decline in forest fragmentation is mainly concentrated in south and northeast China in provinces such as Fujian and Inner Mongolia (Figure S8).

Reduced forest fragmentation, indicated by, e.g., increased forest patch size, implies that afforestation has transformed some forests, which were previously edge forests, into interior forests. The area of these transformed forests (referred to as TFs, Figure 1d, **Methods**) reaches 51.8 M ha, accounting for 57.9% of the afforestation area and 20.6% of the total forest area in China (Table S7). Most TFs are in humid regions (31.2 M ha), with 15.4 M ha in semi‐humid areas and 5.2 M ha in semi‐arid regions (Figure 1f and Figure S24). Spatially, TFs dominate in northeast (e.g., Heilongjiang province) and southwest China (e.g., Yunnan province) (Figure 1f and Figure S8d).

### 2.2 Extra Biogeochemical Effects From the Transformed Forests

**FIGURE 2:**
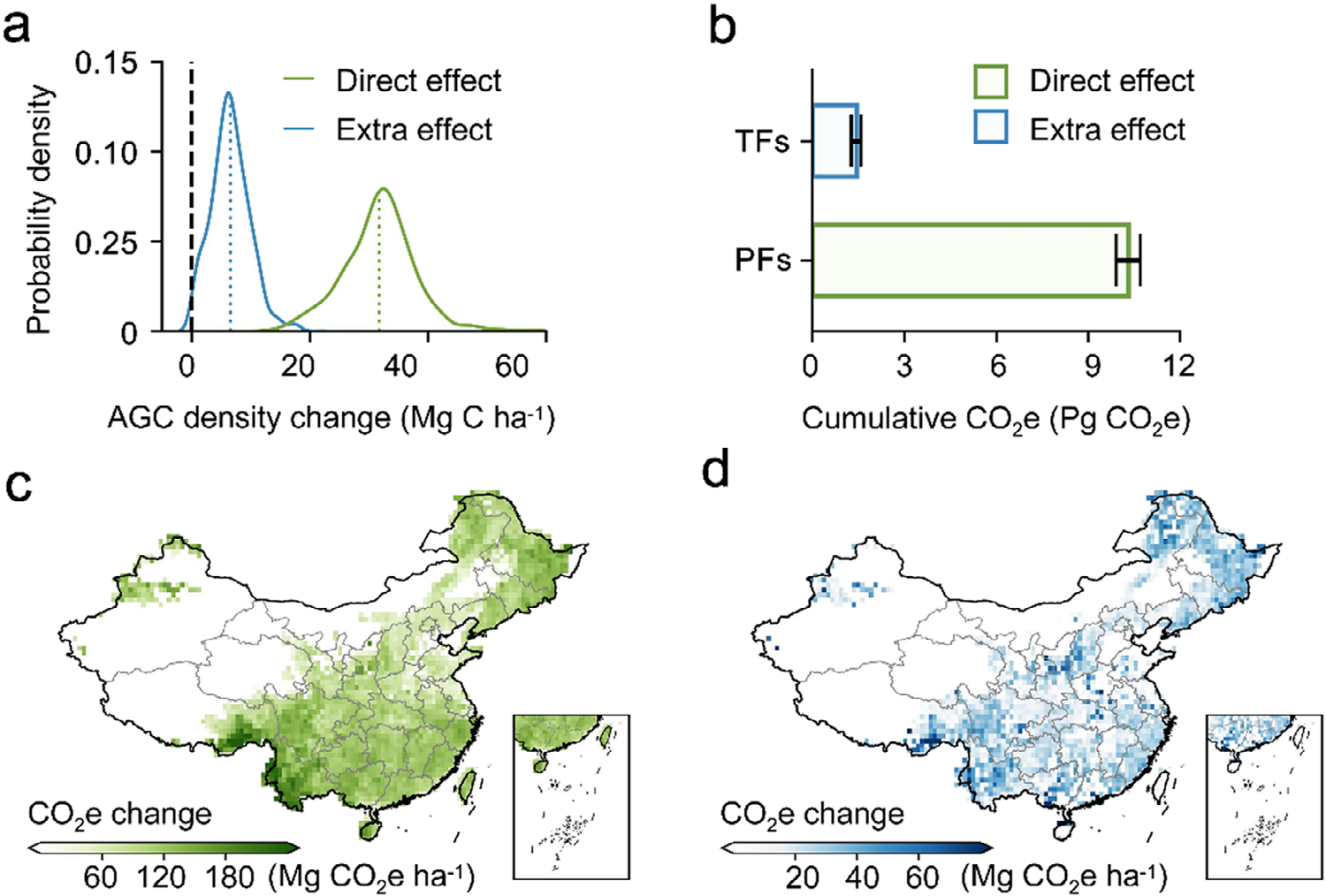
Direct biogeochemical effects from the planted forests (PFs) and extra biogeochemical effects from the transformed forests (TFs) due to afforestation. (a) Kernel density distribution of aboveground biomass carbon (AGC) density change resulting from afforestation, based on the mean value of the three AGB datasets from Santoro et al. [15], ESA CCI [14], and Chen et al. [13]. The colored dotted lines indicate area‐weighted mean values. The direct AGC density change refers to the AGC density difference between the planted forests and their paired open land areas, while the extra AGC density change refers to the AGC density difference between the transformed forests and their paired existing edge forests. (b) Bars represent the mean cumulative CO_2_e change calculated from the three AGB datasets, with error bars showing the standard deviation of cumulative CO_2_e among the three AGB datasets. Positive values denote CO_2_ sequestration from the atmosphere. (c,d) Spatial distribution of CO_2_e change, calculated as the mean value of the three biomass datasets in each 50 km × 50 km grid cell in planted forests (c) and transformed forests (d), respectively.

Compared to nearby open land, the increase in AGC density in PFs is 31.7±6.0 Mg C ha^−1^ (mean ± standard deviation of AGC density across China, based on the mean value of the three AGB datasets from Santoro et al. [15], ESA CCI [14], and Chen et al. [13]). Afforestation also increases AGC densities indirectly by 6.6±3.3 Mg C ha^−1^ in TFs through transforming what were previously edge forests to interior forests (i.e., the AGC density difference between TFs and the paired EFs, after restricting age differences between TFs and EFs to ≤ 5 years; Figure 2a).

We further convert the carbon gains from above‐ and below‐ground biomass into CO_2_e to allow comparison with the biophysical effects (**Methods**). PFs primarily lead to a relatively greater increase in CO_2_e across southwest China, notably in western Yunnan, while relatively lower increases are observed mainly in the North China Plain region, in areas such as Hebei (Figure 2c and Figure S8d). In contrast, regions with an increase in CO_2_e associated with TFs are predominantly located in southwest China (especially Guangxi province) and northeast China (Inner Mongolia and Heilongjiang provinces) (Figure 2d, Figure S8d). The spatial distributions of CO_2_e change for PFs and TFs from each individual biomass dataset (Figure S11) are similar to those shown in Figure 2c,d. By aggregating CO_2_e changes across all regions, we estimate that the cumulative CO_2_e in both PFs and TFs amounts to 11.7±0.5 Pg CO_2_e (mean ± standard deviation across the three aboveground biomass datasets). Specifically, the cumulative CO_2_e associated with PFs in China is estimated at 10.3±0.4 Pg CO_2_e, while that from TFs is 1.4±0.2 Pg CO_2_e, representing 13.6±2.0% of the effect observed in PFs (Figure 2b).

### 2.3 Extra Biophysical Effects From the Transformed Forests

**FIGURE 3:**
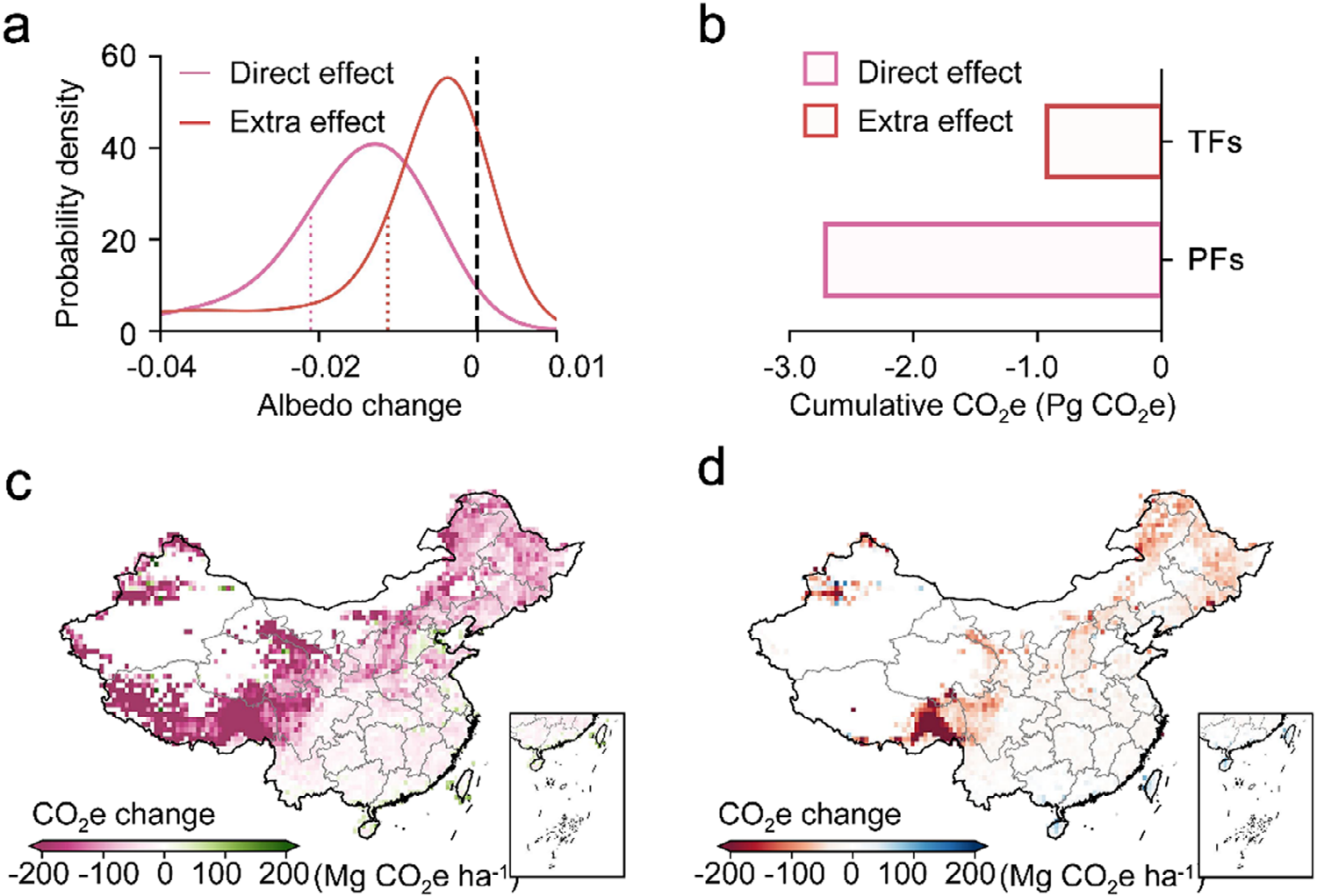
Direct biophysical effects from the planted forests (PFs) and extra biophysical effects from the transformed forests (TFs) due to afforestation. (a) Kernel density distribution of albedo change resulting from afforestation based on the MCD43A3 C6.1 surface albedo data [16]. The colored dotted lines indicate area‐weighted mean values. The direct albedo change refers to the albedo difference between the planted forests and their paired open land areas, while the extra albedo change refers to the albedo difference between the transformed forests and their paired existing edge forests. (b) Bars represent the cumulative CO_2_e changes calculated from the albedo changes, and negative values denote CO_2_ emissions to the atmosphere (warming effect). (c,d) Spatial distribution of albedo changes in each 50 km × 50 km grid cell in planted forests (c) and transformed forests (d), respectively.

In addition to the biogeochemical cooling resulting from CO_2_ removal, as evidenced by increased biomass in PFs and TFs, these forests may also modify surface albedo, potentially inducing biophysical warming or cooling. Compared to nearby open land, the albedo change in PFs is ‐0.02±0.02 (mean ± standard deviation of AGC density across China), based on the MCD43A3 C6.1 surface albedo data [16]. In addition, afforestation indirectly alters albedo in TFs by ‐0.01±0.02 through transforming what were previously edge forests to interior forests, as indicated by the albedo difference between TFs and the paired EFs, after restricting forest age to no more than 5 years (Figure 3a).

The decreased albedo enhances surface absorption of solar radiation, thereby inducing a warming effect (**Methods**). The spatial patterns of both the direct and extra warming effects from PFs and TFs are similar. The largest warming occurs in some semi‐arid regions, such as Tibet, while weaker warming is observed in humid regions, such as Yunnan and Guizhou. In contrast, slight cooling is detected in parts of Guangdong (Figure 3c,d and Figure S8d). Aggregating the albedo‐induced CO_2_e changes across all regions yields a cumulative total of ‐3.6 Pg CO_2_e from both PFs and TFs. Of this total, ‐2.7 Pg CO_2_e is attributed to the direct albedo‐induced warming effect of PFs, while ‐0.9 Pg CO_2_e results from the extra contribution of TFs (Figure 3b).

In addition to the global biophysical climate effects driven solely by albedo changes, PFs and TFs also influence local climate, e.g., land surface temperature (LST), through biophysical processes such as albedo, evapotranspiration and aerodynamic resistance. Daytime LST is 1.6 ± 0.7°C (mean and standard deviation derived from the LST data) lower in PFs compared to nearby open land, and 0.9 ± 0.6°C lower in TFs than in nearby existing edge forests (EFs, Figure S13a). Nighttime differences are smaller, at −0.06°C in PFs and +0.07°C in TFs (Figure S13b), resulting in daily cooling of −0.8 ± 0.4°C in PFs and −0.4 ± 0.3°C in TFs (Figure S13c). These local cooling effects are stronger in northwestern China and weaker in the southeastern regions (Figure S13d,e). Details are provided in Text S6.

### 2.4 Net Effect and the Contributions of Extra Climate Effect

**FIGURE 4:**
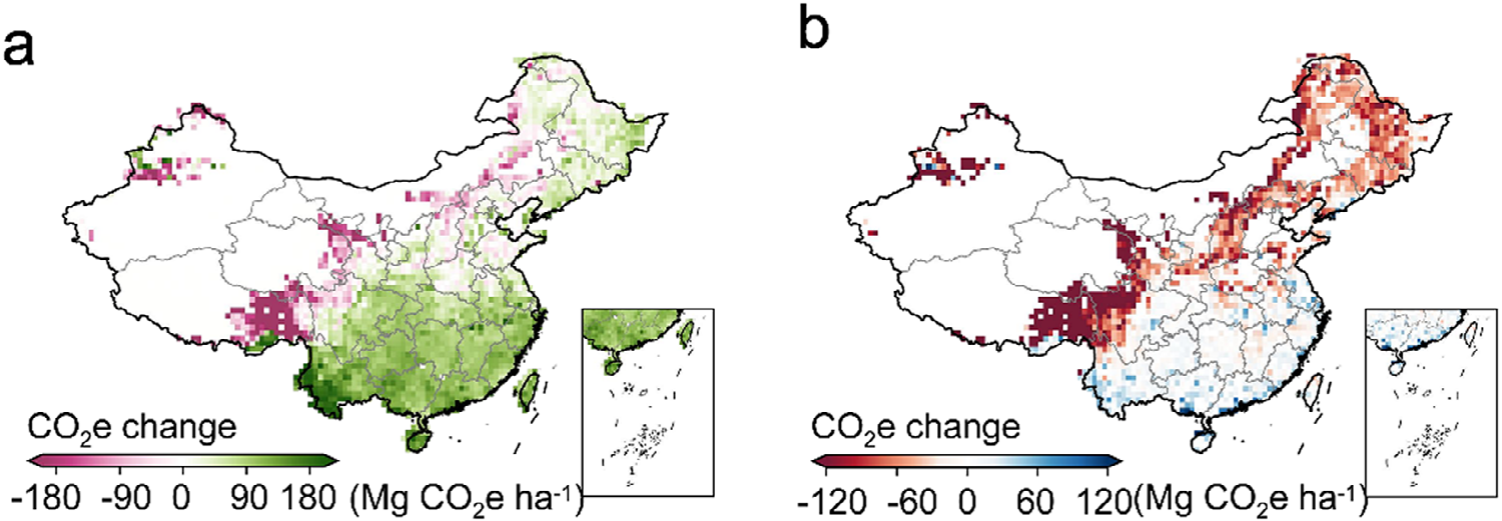
Direct net effects from the planted forests (a) and extra net effects from the transformed forests (b) due to afforestation.

The biophysical warming effects from decreased albedo partially offset the biogeochemical cooling effects from increased biomass, yielding a net climate effect at the global scale. The spatial patterns of this net effect are similar for both PFs and TFs, showing net cooling in the humid regions of Southeast China and net warming in the semi‐arid regions of Northwest China, where afforestation may even exert a negative climate effect due to limited biomass accumulation and albedo‐induced warming (Figure 4). After accounting for both biogeochemical and biophysical effects, the total net CO_2_e change from afforestation amounts to 8.1±0.5 Pg CO_2_e. Of this, PFs contribute a direct CO_2_e change of 7.6±0.4 Pg CO_2_e, while TFs provide an additional 0.5±0.2 Pg CO_2_e, representing an extra 6.6±2.7% of the direct climate benefits delivered by PFs.

Tree species, planted forest subtypes and their age modulate current climate benefits. Across tree species, “Evergreen Fast‐growing” is identified as the optimal species, particularly in southeastern regions where high carbon sequestration and minimal albedo‐induced warming are achieved (Text S11 and Figure S21). Among the three PF subtypes defined by landscape context: isolated PFs (planted forest patches without spatial connection to previously established forests), edge PFs (new trees in the edge zone), and interior PFs (surrounded by previously established forests and edge PFs), interior PFs exhibit the highest AGC density and the greatest decrease in albedo, with these differences becoming more pronounced as forests age (Text S7 and Figures S15–S17). In addition, the age of PFs also influences the timing of TF formation, with TFs formed earlier tending to experience greater AGC increases and stronger albedo reductions. (Text S8 and Figure S18).

We also estimated the potential climate benefits from future afforestation based on the afforestation opportunity map [17]. The total potential climate benefit is estimated at 1.5 Pg CO_2_e, comprising a direct benefit of 1.5 Pg CO_2_e from future PFs and a slightly extra warming (−0.004 Pg CO_2_e) effect from future TFs, likely because these TFs are concentrated in semi‐arid and semi‐humid regions, where limited biomass accumulation and reduced albedo diminish the cooling effect (Text S12 and Figure S22).

## 3 Discussion

Over recent decades, China has implemented a series of large‐scale forest restoration programs such as the Three‐North Shelterbelt Program (1978–2050), the Grain for Green Program (1999–2020), and the Karst Rocky Desertification Control and Restoration Project (2008–2020) [18], aimed at forest conservation and reforestation [19]. These programs have greatly expanded the forest area in China, and reduced forest fragmentation (Text S2, Table S5 and Figure S7). By connecting newly planted forest patches to existing forest patches, ecological restoration projects can increase forest patch size and transform what were previously edge forests into interior forests [20]. The subsequent reduced level of forest fragmentation mitigates the adverse impact of edge effects (Text S4, Figure S10). Consistent with our findings, native forests in the Amazon were found to benefit from secondary forests buffering edge effects and reducing forest fragmentation [11], because the secondary forests can influence the microclimate (e.g., increase relative humidity) and buffer the adverse impact of extreme weather for the surrounding natural forests [21].

The phenomenon of edge degradation due to microclimatic changes after deforestation, such as more frequent droughts, wind turbulence, and fires, has been extensively documented in tropical forests [3, 22]. Globally, remote‐sensing data indicate that edge forests generally exhibit lower average tree cover or AGC density than interior forests, in both tropical and temperate regions [23]. However, there are some exceptions. For example, in the northeastern United States, forest inventory data show that edge forests can grow more vigorously than interior forests due to greater resource availability, such as light [24, 25]. In China, we found that AGC densities are generally lower in the edge forests than in the interior forests, consistent with the majority of global studies. Specifically, the scale of the edge effect is $141_{-31}^{+42}$ m in China based on regional differences nationwide, with a $31_{-6}^{+6}$% reduction in AGC in edge forests compared to interior forests (Text S3, Figure S9). Edge effects are particularly stronger and extend over larger distances in semi‐arid regions than in humid regions (Figure S9), likely due to more pronounced microclimatic stress near forest edges under drier conditions (higher temperatures, lower soil moisture, and stronger winds) [26]. While our findings differ from those in certain temperate regions such as northeastern U.S., where edge forests may grow more vigorously, this discrepancy likely reflects differences in climate, water availability, and other local environmental and management factors. Overall, the edge effects in our study align with the recent global assessment, which reports negative edge effects in approximately 97% of examined regions, with only a few exceptions, such as the Western Siberian grain belt in Russia [6]. These findings are further supported by a recent study in China, which showed gradual increases in normalized difference vegetation index (NDVI), canopy cover, and other forest structure metrics from edges to the interior [27].

The datasets and methods used in this study may involve several sources of uncertainty (Text S13). AGB datasets may underestimate biomass in areas with high AGB [15], although the overall patterns remain consistent across multiple datasets (Tables S10 and S11). Differences in spatial resolution and data sources among remote‐sensing products may also introduce minor uncertainties. Our estimates of AGC density and albedo changes rely on a space‐for‐time method. These estimates reflect realized cumulative climate effects over the ~1980–2015 period, representing the integrated impact of existing forest age distributions rather than the theoretical maximum potential at forest maturity. They should therefore be interpreted as climate benefits achieved to date, rather than the eventual benefits that may be reached under continued forest growth. Within each 50 km × 50 km grid cell, we assumed that climate conditions and forest management are broadly comparable between TFs and their paired EFs. Residual differences may persist but are expected to be relatively small. In this study, the direct climate effects of PFs were compared with “open land” defined as non‐forest reference conditions obtained by masking out forested areas. While this provides a consistent baseline, we note that different open land types may vary in albedo, carbon storage, and climate feedbacks, thereby introducing some additional uncertainty. However, because the source areas of PFs are also heterogeneous, using an aggregated open‐land reference is likely to be more robust and representative at the national scale than adopting a single open‐land type. It should be noted that the TF–EF comparison reflects the climate benefits associated with improved forest configuration, rather than providing direct evidence of the temporal dynamic effects of afforestation. Long‐term monitoring and experimental studies are therefore needed to capture process‐based changes in forest connectivity and local microclimate. In addition, potential interactions between biogeochemical and biophysical processes (e.g., carbon‐driven canopy changes influencing evapotranspiration or surface roughness) were not explicitly represented. Our global biophysical estimates are derived solely from satellite‐observed albedo changes and are treated independently from biogeochemical pathways, consistent with previous large‐scale assessments [2]. However, real ecosystems may involve additional feedbacks that warrant further investigation.

Our study goes beyond the effects typically considered in forest management and policymaking, emphasizing the extra climate benefits arising from edge‐to‐interior transformations due to reduced forest fragmentation. These benefits account for 6.6±2.7% of the direct climate benefits related to biochemical cooling effect from increased biomass and biophysical warming effect from decreased albedo in newly planted forests, equivalent to the climate benefits provided by an extra 5.9 M ha of newly planted forests. By the 2060s, the potential afforestation area in China is estimated at 78 M ha, based on high‐confidence potential forest distributions that do not overlap existing forest, agricultural land, or human settlements [17]. However, the achievable afforestation area may be lower due to conflicts with socioeconomic developments and water supply. Our study provides new insights into the scientific planning of afforestation on limited land to maximize climate benefits. Future afforestation should therefore prioritize connecting small forest patches into larger, more contiguous patches to enhance edge‐to‐interior transformation, select tree species that deliver net climate benefits, and consider their seasonal dynamics so as to optimize carbon sequestration while minimizing biophysical warming from albedo reduction. Particular attention is needed in arid and semi‐arid regions, such as Qinghai and Tibet, where afforestation may otherwise lead to adverse climate effects or exacerbate local water stress. In addition to total afforested area, performance indicators, e.g., the edge‐to‐interior conversion proportion, can be introduced to better evaluate the actual climate and ecological benefits of afforestation projects in the future. Moreover, metrics such as the net CO_2_‐equivalent benefit per unit area and the proportion of transformed (edge‐to‐interior) forests could be embedded in the design and assessment of large‐scale restoration programs (e.g., the Three‐North Shelterbelt Program and the Grain for Green Program), thereby linking afforestation and landscape planning more explicitly to national carbon‐neutrality targets and regional climate‐adaptation goals.

## 4 Methods

### 4.1 Forest Extent Identification and Accuracy Validation

We employed data with a high spatial resolution to delineate the forest extent. Specifically, we used the global forest management dataset for 2015 from Lesiv et al. [12], which was georeferenced using the WGS84 coordinate system and has a spatial resolution of 0.00099° (~100 m). We first extracted forest extent data within the terrestrial borders of China using the administrative boundaries. Forest extent data were then classified into three classes: natural forests, planted forests (PFs) and others, following the criteria outlined in Table S1. Based on the definitions of Lesiv et al. [12], natural forests refer to naturally regenerating forests, including both those without any signs of management and those with signs of management. PFs refer to planted forests, including those with a rotation period greater than 15 years and those with a rotation period of 15 years or less. The forest extent map is shown in Figure S4. Finally, the accuracy of forest areas was evaluated at field (Figure S5 and Table S3), provincial (Figure S6) and national scales (Table S4), with all evaluations yielding satisfactory results (Text S1).

### 4.2 Changes in Forest Fragmentation Due to Afforestation

Afforestation can influence forest fragmentation through multiple spatial processes, including expansion of existing patches and connection of isolated patches [28] (Figure S1). Impacts of different afforestation types on fragmentation may be reflected by different fragmentation indices. For example, when PFs only expanded around a single forest patch (Figure 1d) without connecting multiple forest patches, some fragmentation indices may not change (e.g., patch density), but some other fragmentation indices (e.g., mean patch area) may change. We thus used multiple fragmentation indices in our analyses, including edge density (Equation 1), patch density (Equation 2), and mean patch area (Equation 3).

To quantify these effects, we divided the study area into 50 km × 50 km grid cells, each assumed to have uniform climate characteristics and forest management practices [29]. Within each grid cell, forest pixels were aggregated into distinct forest patches using a four‐neighbor algorithm [3]. The three forest fragmentation metrics, were then calculated both before (prior to ~1980) and after (by 2015) afforestation [10], representing the cumulative forest changes over this ~35‐year period. The forest fragmentation metrics before afforestation were calculated based on the natural forest/non‐forest binary maps. The term “non‐forest” includes the pixels of planted forests because these pixels were not afforested before afforestation. Subsequently, the forest fragmentation metrics after afforestation were calculated based on forest/non‐forest binary maps. Afforestation refers to planted forests, therefore, the term “forest” encompasses both natural forests and planted forests after afforestation. Finally, we calculated the differences in three forest fragmentation metrics between before (prior to ~1980) and after (by 2015) afforestation. An increase in edge density and patch density and a decrease in mean patch area indicate increased forest fragmentation, and vice versa.

(1) $$ED=\frac{\sum_{i=1}^{N}EL_{i}}{A}\;$$

(2) $$PD=\frac{N}{A/100}\;$$

(3) $$MPA=\frac{\sum_{i=1}^{N}A_{i}}{N}$$

where *ED*, *PD*, and *MPA* are the edge density (m ha^−1^), patch density (per 100 ha), and mean patch area (ha), respectively. *EL_i_* is the edge length of patch *i* in meters (m), *A_i_* is the area of patch *i* in hectares (ha), *N* is the total number of patches, and *A* is the total landscape (50 km × 50 km) area in hectares (ha).

### 4.3 Classification of Forest Types Based on Landscapes

Forests were classified into four categories: planted forests (PFs), transformed forests (TFs) from edge to interior, existing edge forests (EFs, i.e., forests consistently located at the edge zone since ~1980), and interior forests (IFs) (Figure 1d; Figures S1 and S3). PFs and TFs were defined to provide direct and extra climate benefits, respectively (Figure S2). The specific classification criteria are as follows.

i) PFs were derived from the reclassified forest extent map [12] (Figure S4a and Table S1). PFs were further classified into three types based on the landscape: isolated PFs (planted forest patches without spatial connection to previously established forests, where the previously established forests refer to the combination of I and II before afforestation in Figure 1d); edge PFs (new trees in the edge zone); and interior PFs (surrounded by previously established forests and edge PFs). The classification criteria and results are detailed in Text S7, Figures S3 and S15.

ii) The relationship between biomass and distance from the forest edge was employed to fit the scales of edge effects within each 50 km × 50 km grid cell [5, 30] (Equation 4). This approach allowed us to derive a distinct edge effect scale for each grid cell rather than applying a single, uniform value across the study area. Based on these grid cell‐specific estimates, the median edge effect scale in China was 141 m, with the 25th and 75th percentiles being 110 and 183 m, respectively. Sensitivity analyses conducted at three alternative resolutions yielded consistent results (Text S3, Table S6). Natural‐forest pixels with distances to the forest edge of less than the scale were classified as EFs, while those with greater distances were classified as IFs.

(4) $$AGB=\theta_{1}\;-\theta_{2}\times e^{-\theta_{3}\times DIS}$$

where *AGB* represents the aboveground biomass (Mg ha^−1^), *θ_1_* is the fitted maximum *AGB* in the interior forests, *θ_2_* and *θ_3_* are fitting parameters, *e* is Euler's number, and *DIS* is the distance to the non‐forest edge (m). The scale was defined to be the distance at which *AGB* equals 90% of the maximum *AGB* in the interior forests.

iii) TFs refer to forest pixels located in the edge zone (edge distance < scale) of a forest patch before afforestation, which subsequently become interior forests due to the establishment of planted forests (Figure 1d and Figure S1). We obtained the natural forest/non‐forest binary maps before afforestation and then the fitted scale of edge effect within each 50 km × 50 km grid cell was used to identify the edge forest pixels before afforestation. For each edge forest pixel before afforestation, we checked whether there were PF pixels within the edge distance after afforestation. If so, the pixel is designated as TF (Figure 3b).

### 4.4 Estimation of the Biogeochemical Climate Benefits

The biogeochemical climate benefits were quantified by calculating the biomass accumulation resulting from afforestation, considering both above‐ and below‐ground biomass. This biomass accumulation was then converted to CO_2_e by multiplying by the mass ratio of CO_2_ to carbon (Equations 5 and 6). The three AGB datasets were Santoro et al. [15] for 2010, ESA CCI [14] for 2017, and Chen et al. [13] for 2015, with spatial resolutions of 25 m, 100 m, and 1 km, respectively.

(5) $$CO_{2}e_{biomass}=\sum_{i=1}^{n}\left(\Delta CO_{2}e_{i,biomass}\times A_{i}\right)\;$$

(6) $$\Delta CO_{2}e_{i,biomass}=\left(\Delta AGB_{i}+\Delta BGB_{i}\right)\times \varepsilon \times \frac{44}{12}$$

where *CO_2_e_biomass_* is the total biogeochemical climate benefit of afforestation for the study region (Mg CO_2_e). *∆CO_2_e_biomass_* is the grid cell‐level (50 km × 50 km) CO_2_e change due to biomass change (Mg CO_2_e ha^−1^). ∆*AGB_i_* and ∆*BGB_i_* are the changes in above‐ and below‐ground biomass (Mg ha^−1^). For the direct climate effect, ∆*AGB_i_* and ∆*BGB_i_* represent the above‐ and below‐ground biomass in PFs, and *A_i_* is the PFs area in the *i_th_* grid cell (ha); For the extra climate effect, they represent the biomass difference between TFs and their paired EFs, and *A_i_* is the TFs area in the *i_th_* grid cell (ha). The mean age difference between TFs and their paired EFs is limited to within 5 years in each 50 km × 50 km grid cell. A root‐to‐shoot ratio of 0.26 is used to estimate belowground biomass from aboveground biomass (*ΔBGB_i_* = 0.26 × *ΔAGB_i_*) for temperate forests [31]*. ε* is the conversion coefficient from AGB to AGC density, and *ε* is 0.5 in this study [32].

### 4.5 Estimation of the Biophysical Climate Benefits

Changes in surface albedo can modify Earth's radiative balance, thereby affecting global climate by altering the amount of energy absorbed or reflected by the planet [33]. To assess these effects, we compared the biophysical impact of albedo changes with the biogeochemical impact of changes in atmospheric CO_2_, and we converted albedo changes into CO_2_‐equivalent units following Hasler et al. (2024) [2] to allow a direct comparison with changes in the CO_2_‐driven forcing. Details are provided in (Equations (7), (8), (9), (10)). In addition, we quantified local land surface temperature (LST) changes to capture regional responses. Unlike albedo‐driven effects, these local LST changes, driven primarily by variations in evapotranspiration and energy fluxes, represent regional climate responses rather than global‐scale effects [34]. LST differences between TFs and EFs were calculated only when their DEM difference was less than 100 m, as detailed in Text S6 and Figures S13 and S14. We used 2015 MCD43A3 surface albedo data (500 m) [16], Landsat LST data (30 m) [35], and MODIS LST data (1 km) [36], along with NASADEM digital elevation data at 30 m resolution.

(7) $$CO_{2}e_{albedo}=\sum_{i=1}^{n}\left(\Delta CO_{2}e_{i,albedo}\times A_{i}\right)$$

(8) $$\;\Delta CO_{2}e_{i,albedo}=\frac{M_{CO_{2,global}}^{albedo}}{A_{global}}$$

(9) $$M_{CO_{2,global}}^{albedo}=\frac{RF_{i}}{\frac{dF}{dCO_{2}}\times \gamma }$$

(10) $$RF_{i}=\frac{\sum_{j=1}^{12}\left(K_{i,j}\times \Delta \alpha_{i,j}\times D_{j}\right)}{365}$$

where *CO_2_e_albedo_* is the total biophysical climate effect of afforestation through albedo change for the study region (Mg CO_2_e). ∆*CO_2_e_i,albedo_* is the CO_2_e climate effect due to albedo change for the *i_th_* grid cell (50 km × 50 km) (Mg CO_2_e ha^−1^). *RF_i_* is the time‐weighted mean‐annual top‐of‐atmosphere (TOA) radiative forcing (W m^−2^). *K_i_* is the spatially and temporally explicit monthly surface‐albedo radiative kernel (W m^−2^ per unit albedo), derived from the radiative kernel dataset based on the ECMWF Reanalysis v5 (ERA5) [37]. *∆α_i_* is the monthly albedo change due to afforestation (unitless). For the direct climate effect, Δα_*i*_ represents the albedo difference between PFs and their paired open land, defined as all areas not classified as forest after masking out forested areas [12], and *A_i_* is the PFs area in the *i_th_* grid cell (ha); For the extra climate effect, it represents the albedo difference between TFs and their paired EFs, and *A_i_* is the TFs area in the *i_th_* grid cell (ha). The mean age difference between TFs and their paired EFs is limited to within 5 years in each 50 km × 50 km grid cell to avoid the impacts of age difference. *dF/dM_CO2_* is the radiative efficiency of CO_2_ (W·m^−2^ per kg CO_2_), which quantifies the change in top‐of‐atmosphere radiative forcing caused by a unit increase in atmospheric CO_2_ mass. It was derived from the empirical relationship between CO_2_ concentration and radiative forcing, given by: *ΔF = 5.35ln(C/C_0_​*), where *ΔF* is the radiative forcing (W·m^−2^) resulting from a change in atmospheric CO_2_ concentration from a pre‐industrial level C_0_ (∼270 ppm) to the current level C (~400 ppm) [2]. Using the conversion 1 ppm ≈ 7.81 × 10^12^ kg CO_2_, this yields *dF/dM_CO2_* ≈ 2.45 × 10^−15^ W m^−2^ per kg CO_2_. *A_global_* is the Earth's surface area (ha). *γ* is the 100‐year atmospheric retention fraction of CO_2_, set to 0.47, based on recent empirical estimates of the airborne fraction [38].

### 4.6 Proportion of Extra Climate Benefits to the Direct Climate Benefits

We further quantified the contribution of the extra climate benefits relative to the direct climate benefits, considering both biomass‐driven carbon sequestration and albedo‐induced carbon emissions. It should be noted that our estimates represent realized cumulative climate effects up to the survey period (~2015), rather than the theoretical maximum potential at forest maturity. By employing a space‐for‐time substitution approach centered on remote‐sensing datasets from ~2015, we captured the net climate impacts of afforestation initiated since the 1980s. Consequently, the reported CO_2_e changes reflect the integrated benefits over a ~35‐year window, accounting for the existing stand age distribution and landscape conditions, rather than the full lifetime effects of forests after maturity.

(11) $$P=\frac{CO_{2}e_{biomass,extra}+CO_{2}e_{albedo,extra}}{CO_{2}e_{biomass,direct}+CO_{2}e_{albedo,direct}}\;\times 100$$

where *P* (%) is the contribution of the extra climate benefits relative to the direct climate benefits. *CO_2_e_biomass,extra_* (Mg CO_2_e) and *CO_2_e_albedo,extra_* (Mg CO_2_e) are the extra biogeochemical climate benefits and the extra biophysical climate benefits from TFs, respectively. *CO_2_e_biomass,direct_* (Mg CO_2_e) and *CO_2_e_albedo,direct_* (Mg CO_2_e) are the direct biogeochemical climate benefits and the direct biophysical climate benefits from PFs, respectively.

## Author Contributions

**W.L**. designed the research. **N.M**. performed the analysis. **N.M**. and **W.L**. wrote the draft. **P.C**., **M.B**., **X.T**., **Y.Y**., **G.S**., **W.D**., **L.Z**., **Y.L**., **M.S**., **M.H**., **X.Z**., **J.Z**., **N.W**., **D.T**., **C.W**., **W.D**., **S.T**., and **B.P**., contributed to the interpretation of the results and the writing of the paper.

## Conflicts of Interest

The authors declare no conflicts of interest.

## Data Availability

All the datasets used in this study are publicly available. The forest management map (2015, 100 m) is available at https://doi.org/10.5281/zenodo.5879022 [12]. The three AGB datasets can be downloaded from https://doi.org/10.1594/PANGAEA.894711 (2010, 25 m) [15], https://catalogue.ceda.ac.uk/uuid/af60720c1e404a9e9d2c145d2b2ead4e (2017, 100 m) [14], https://doi.org/10.6084/m9.figshare.21931161.v1 (2015, 1 km) [13]. The surface albedo data (2015, 500m) is the MCD43A3 data, which can be downloaded from https://doi.org/10.5067/MODIS/MCD43A3.061 [16]. The forest age map (2020, 30 m) is available from https://doi.org/10.6084/m9.figshare.21627023.v7 [39]. The tree species map (1:1,000,000) for China from the forest inventory conducted between 2013 and 2017 is available at http://www.doi.org/10.12041/geodata.43370179401687.ver1.db. The potential forest distribution map (2060, 1 km) is available at https://zenodo.org/records/8297679 [17]. The Landsat LST dataset (2015, 30 m) [35], MODIS LST dataset (2015, 1 km) [36], and DEM data (NASADEM: NASA NASADEM Digital Elevation 30 m) are available from Google Earth Engine (https://earthengine.google.com/). The radiative kernel dataset is available from https://doi.org/10.17632/vmg3s67568 [37].
